# Identifying the needs of penile cancer sufferers: A systematic review of the quality of life, psychosexual and psychosocial literature in penile cancer

**DOI:** 10.1186/1471-2490-9-8

**Published:** 2009-08-08

**Authors:** Satish B Maddineni, Maurice M Lau, Vijay K Sangar

**Affiliations:** 1 Department of Urology, The Christie Hospital, Manchester, UK; 2 Department of Urology, University Hospital of South Manchester, Manchester, UK; 3 Division of Cancer Studies & Imaging, University of Manchester, Manchester, UK

## Abstract

**Background:**

Penile cancer is an uncommon malignancy with an incidence of 1 per 100,000. Conservative and radical treatments can be disfiguring and may have an impact on sexual function, quality of life (QOL), social interactions, self-image and self-esteem. Knowledge of how this disease affects patients is paramount to developing a global, multi-disciplinary approach to treatment.

**Methods:**

A Medline/PubMed literature search was conducted using the terms "sexual function penis cancer"; "quality of life penis cancer" and "psychological effects penis cancer" from 1985 to 2008. Articles containing quantitative data on QOL, sexual function or psychological well-being were included.

**Results:**

128 patients from 6 studies were included. 5 studies contained retrospective data whilst 1 study collected prospective data on erectile function. In the 6 studies 13 different quantitative tools were used to assess psychological well-being, QOL and sexual function. The General Health Questionnaire (GHQ) showed impaired well-being in up to 40% in 2 studies. Patients undergoing more mutilating treatments were more likely to have impaired well-being. The Hospital Anxiety and Depression Score (HADS) demonstrated pathological anxiety up to 31% in 2 studies. 1 study used the Diagnostic and Statistical Manual of Mental Disorders of psychiatric illness (DSM III-R) with 53% exhibiting mental illness, 25% avoidance behaviour and 40% impaired well-being. 12/30 suffered from post-traumatic stress disorder. The IIEF-15 was the commonest tool used to assess sexual function. The results varied from 36% in 1 study with no sexual function to 67% in another reporting reduced sexual satisfaction to 78% in another reporting high confidence with erections.

**Conclusion:**

The treatment of penile cancer results in negative effects on well-being in up to 40% with psychiatric symptoms in approximately 50%. Up to two-thirds of patients report a reduction in sexual function. This study demonstrates that penile cancer sufferers can exhibit significant psychological dysfunction, yet no standardised tools or interventional pathways are available. Therefore, there is a need to identify and assess adequate tools to measure psychological and sexual dysfunction in this group of patients.

## Background

Cancer of the penis is a relatively uncommon malignancy in Western countries with a reported incidence of 1 per 100,000 in Europe [1]. Social and cultural habits influence the incidence of disease which is related to exposure to the human papilloma virus (HPV) types 16 and 18, socio-economic factors, chronic irritation, phimosis and smoking [2]. There are a range of treatments available for localised early disease such as topical 5-Flurouracil therapy, laser therapy, glans resurfacing and glansectomy with reconstruction. For more advanced disease partial or total penectomy with or without reconstruction or radiotherapy is advocated. Surgical treatment is generally accepted as the gold standard for high grade and high stage disease [3,4]. In addition, patients with inguinal node disease undergo regional lymphadenectomy which can be debilitating with complications such as lymphoedema, infection and wound dehiscence occurring in up to 40% of individuals.

All treatments may be disfiguring and this may have an impact on the patient's sexual function, quality of life (QOL), social interactions, self-image and self-esteem. Knowledge of how this disease affects patients will provide health professionals and organisations with the means of identifying interventions and resources for this group of patients.

The purpose of this review is to examine the current literature on the effects of curative penile treatment on sexual function, quality of life and psychological well being.

## Methods

An extensive Medline/PubMed literature search was conducted using the terms "sexual function penis cancer"; "quality of life penis cancer" and "psychological effects penis cancer" from 1985 to 2008. The search was undertaken independently by two of the authors (SBM & VKS). Abstracts were retrieved and sorted for eligibility. Those abstracts containing quantitative data on quality of life, sexual function or psychological well being were included. All non-English studies, case reports, studies reporting surgical and survival outcomes only, reviews, editorial comments and those studies containing qualitative data only were excluded from the review.

Full length articles were retrieved and reviewed to ensure inclusion criteria were met. Any studies containing repeat patients from the same institute were excluded. Data were entered onto a Microsoft Excel spreadsheet for analysis.

Statistical analysis was considered in the form of weighted and un-weighted means for those studies that contained similar quantitative instruments and where presentation of data and study design allowed.

## Results

In total, 437 studies were found in the three searches. Abstract review revealed that 10 studies fulfilled the inclusion criteria of which 2 articles were duplicated and subsequently excluded. The remainder of the abstracts retrieved in the 3 searches did not relate directly to penile cancer patients, focussed on only the surgical aspects of penile cancer; the viral aetiology of penile cancer; case reports of isolated experiences with penile cancer and overviews/reviews of the aetiology and management of penile cancer. Therefore, a total of 8 full journal articles were assessed. One article contained repeat patients from the same institution whilst another contained no quantitative data for the purpose of this study. Hence 6 full articles were suitable for the purpose of this review (Table 1).

**Table 1:** Studies included in systematic review

**Author**	**Bibliography Reference**	**Year Study Published**	**Patient Numbers**	**Treatment Used**
Ficarra	[5]	2000	16	Partial penectomy
Romero	[6]	2005	18	Partial penectomy
D'Ancona	[7]	1997	14	Partial penectomy
Windahl	[8]	2004	36	Laser Therapy
Opjordsmen	[9]	1994	30	All Treatments
Gulino	[10]	2007	14	Glansectomy/Partial penectomy

### Patients

In total, there were 128 patients from 6 studies (range 14 to 36) with a mean follow-up that ranged from 11.5 to 80 months. All but 1 study contained only penile cancer patients. The treatments received within each study ranged from partial penectomy only, partial penectomy and glansectomy only, laser therapy only and all treatments (Table 1).

### Study designs

5 studies contained retrospectively collected data whilst 1 study collected pre-treatment data on erectile function prior to disease. The same study also asked about QOL prior to and during disease. Therefore no single study was entirely prospective. The control groups in each study varied and included internal retrospective, internal prospective, a mixture of the previous two or external retrospective controls (Table 2). The study by Ficarra et al (2000) used patients who had undergone treatment for benign prostatic hyperplasia (BPH) as the control group [5]. This group obviously represents a separate cohort of patients with limited parallels to those suffering with penile cancer and therefore significantly limits the comparative value of the study.

**Table 2:** Study design and mean follow-up in months

**Author**	**Bibliography Reference**	**Year Study Published**	**Study Design**	**Control Group**	**Mean Follow-up (months)**
Ficarra	[5]	2000	Retrospective	Post BPH treatment	69
Romero	[6]	2005	Retrospective	Retrospective	23.5
D'Ancona	[7]	1997	Retrospective	Nil	11.5
Windahl	[8]	2004	Retrospective	Retrospective	36
Opjordsmen	[9]	1994	Retrospective	Nil	80
Gulino	[10]	2007	Prospective/Retrospective	Prospective/Retrospective	13

In the 6 studies there were a total of 13 different quantitative tools used including self made questionnaires (Table 3). These tools were administered in a variety of ways including self administered by the patients, semi-structured interviews, general interviews and a mixture of these methods (Table 3). In some cases similar tools were administered in different ways in separate studies.

**Table 3:** Parametric tools used in each of the studies

**Author**	**Bibliography Reference**	**Year Study Published**	**Parametric Tools used in Study**	**Administration of assessment tools**
Ficarra	[5]	2000	ECOG GHQ HADS	Self Completion Single time point
Romero	[6]	2005	IIEF-I5	Interview Single time point
D'Ancona	[7]	1997	OSFQ SPQ GHQ HADS	Semi-structured interview Single time point
Windahl	[8]	2004	Self made sexual activity Self made sexual function Ed IIEF-II LiSat-11	Semi-structured interview Single time point
Opjordsmen	[9]	1994	Interview DSM III-R PAIS GHQ EORTC QLQ	Semi-structured interview Self Completion Single time point
Gulino	[10]	2007	IIEF-15 Bigelow & Young	Self Completion Single time point

### Statistical analysis

As no two studies contained identical measures performed and presented in the same way no statistical comparison was possible between studies.

### Psychological Well Being/Stress

The studies used a number of measures for psychological well being. The General Health Questionnaire (GHQ) showed impaired well being in 37.5% and 40% of patients in the Ficarra [5] and Romero [6] studies respectively whilst no patients exhibited such impairment in the D'Ancona [7] study (Table 4). In addition, the Ficarra study showed that patients having more mutilating treatments were more likely to have impaired well being. All three studies were retrospective and used different cut-off values to define significant impairment.

**Table 4:** Results of psychological well being/stress

**Parametric Assessment Tool Used** **(Study)**	**Study Group** **(%)**	**Control Group** **(%)**	**Comment**
**ECOG**			
Ficarra	0	n/a	Performance status
**GHQ**			
Ficarra	37.5*	9	Pathologic cut off >3
D'Ancona	0	n/a	Pathologic cut off 3/4
Opjordsmen	40	n/a	% ≥ 5
**HADS**			
Ficarra	31*	2	Pathological anxiety
D'Ancona	0	n/a	Cut off 8/9
**SPQ**			
D'Ancona	No data		
**PAIS**			
Opjordsmen	24.5	n/a	Intrusion & avoidance ≥ 9
**Bigelow & Young**			
Gulino	16*	30	Feelings of pleasantness
	37*	18	Feelings of unpleasantness
	16*	4	Relation with family/partner
	22	20	Social relations [during disease (control gp) vs post operative] (No differences between before disease vs post op)
**EORTC QLQ**			
Opjordsmen	3.1	n/a	Overall well being
	2.1	n/a	Overall sexual well being (4 best – 0 worst)
**Interview DSM III-R**			
Opjordsmen	53.3	n/a	Presence of mental symptoms

(* indicates significant result as reported in study).

The Hospital Anxiety and Depression Score (HADS) was used in the D'Ancona and Romero studies and demonstrated pathological anxiety in 0 and 31% of patients respectively. This variation is likely to be due to differing definition levels of significance and methods of administration and reporting.

The Ficarra study used psychiatric interviews and assessed patients in relation to the Diagnostic and Statistical Manual of Mental Disorders of psychiatric illness (DSM III-R). It showed that 53.3% of patients exhibited signs and symptoms of mental illness with 2 out of 30 patients exhibiting post-traumatic stress disorder (PTSD). Nearly 25% showed signs of avoidance behaviour and 40% exhibited impaired well being as measured by GHQ; the latter suggesting that 12 out of 30 patients had signs of PTSD. Interestingly the same authors also used the European Organisation for Research and Treatment of Cancer quality of life questionnaire (EORTC QLQ C30) to assess well being at a level of 3.1 (4 best to 0 worst), suggesting mild to moderate impact on well being.

Therefore, although correlation between QOL outcome measures is generally limited all tools used in the Ficarra study exhibit a degree of consistency. However, the sensitivity of each outcome measure can be varied depending on the factors assessed, the manner in which they are assessed and definitions of significance with each tool used. In the case of the Ficarra study there is reasonable transferable correlation and indeed sensitivity between the GADS and GHQ-12 questionnaires. However, the sensitivity of these questionnaires in relation to penile cancer sufferers is debatable. In the D'Ancona study although there is no stated effect of surgery on quality of life/psychological well being there is again consistency between the different outcome measures. However, the lack of effect is a reflection of the definition of significance used for both outcome measure tools in the study.

The Bigelow & Young questionnaire used in the Gulino study showed that scores for feelings of unpleasantness reduced from 30 pre-operatively to 16 following treatment. In addition, scores for relationships with family/partner improved from 4 to 16. The study scored patients before disease (retrospectively), during disease (retrospectively) and following treatment. There was no significant difference in scores between before disease and after treatment, but there was a significant improvement between disease period and post treatment scores, suggesting a positive impact from treatment.

### Sexual Function

The International Index of Erectile Function Questionnaire (IIEF-15) was the commonest tool used for assessing sexual function (the Romero, Windahl and Gulino studies) (Table 5). In the Windahl study, only 10 patients out of 36 completed the IIEF-15 [8]. 6 of these were not sexually active whilst 4 scored mild to moderate erectile dysfunction. However, using the Fugl-Meyer Life Satisfaction Check List score (LiSat-11), 50% were shown to be satisfied with their sex life. In the Romero study, 14 out of 18 patients (78%) had high or very high confidence in obtaining erections, whilst 3 out of 18 (17%) had no orgasm, 6 out of 18 (33%) had reduced sexual desire and 12 out of 18 (67%) had reduced overall satisfaction compared to pre-treatment [9]. It should be noted that patients in this study underwent less mutilating treatment in the form of laser therapy. The Gulino study observed scores between before disease (retrospective) and post treatment and showed no significant difference [10].

**Table 5:** Results of sexual function

**Assessment Tool used** **(Study)**	**Study Group (%)**	**Control Group (%)**	**Comment**
**IIEF-15**			
Romero	19.39*	29.56	Erectile function (14/18 with high confidence)
	7.67*	9.94	Orgasm (3/18 no orgasm)
	7.61*	8.89	Sexual desire (6/18 reduced post-op)
	6.89*	12.67	Intercourse satisfaction (6/18 no intercourse)
	6.11*	8.61	Overall satisfaction (12/18 reduced satisfaction post-op)
Windahl	n/a	n/a	10 had ED, 6 had no intercourse, 1 mild ED, 3 moderate ED.
Gulino	21	22	Erectile function before disease vs post-op function at 12 months
	13	12	Orgasm " " " " "
	8	9	Libido " " " " "
**LiSat-II**			
Windahl	50	n/a	Proportion satisfied with sex life
**Self made sexual activity**			
Windahl	28.6	14.3	Proportion with reduced activity
**Self made sexual function**			
Windahl	16.7	22.2	Proportion with decreased desire
**ED**			
Windahl	16.7	8.3	Proportion decreased

(* indicates statistically significant difference as reported in study)

D'Ancona's group used the Overall Sexual Functioning Questionnaire (OSFQ) and showed that almost 36% of patients had no sexual function or moderately to severely reduced sexual function [6].

Ficarra et al showed that patients had moderate sexual function scores with a mean of 2.1 (4 = best function; 0 = worst function) across all treatment groups, but those patients who underwent more mutilating treatment had lower scores (1.3 and 1.0 for partial and total penectomy respectively) [5].

## Discussion

The purpose of this systematic review was to establish the overall effects of penile cancer and its treatment on psychosexual, social and quality of life experiences.

The data suggest a mixed impact on these issues, with negative effects on well being in up to 40% and psychiatric symptoms in approximately 50%, with signs of post-traumatic stress disorder in almost the same proportion. However, other studies have shown that treatment may improve feelings of unpleasantness and poor relationships. Furthermore up to two-thirds of patients exhibit some form of negative impact on their sexual function.

Importantly, no statistical analysis was possible due to the lack of well designed studies (level III evidence at best) and therefore the data should be interpreted with caution. Currently it is possible to infer that penile cancer and its treatment can affect sexual function, psychological well being, quality of life, and may also result in post-traumatic stress disorder. In cases of negative impact, these issues are likely to provide the need for significant interventions. However the review does not provide any bearing as to which types of tools can be used to identify such problems and how such problems can be avoided or resolved.

The limitations of this study lie in the poor quality articles included in the analysis. The majority of studies used retrospective data collection from small numbers of patients in single units, using different measuring tools. In addition the means of delivering the study tools differed from self administration, interview and semi-structured interview. In studies that had similar measuring tools, authors subsequently chose a different means of analysing raw data and with varying cut-off points. These are likely to introduce reporting bias in most of the studies. Furthermore the groups of patients studied varied in stage of disease and type of treatment administered, which is likely to affect the amount of psychological or sexual dysfunction experienced.

Depression and suicidal ideation is not uncommon in cancer sufferers. Schairer et al [11] showed, from the SEER database, that the risk of suicide amongst breast cancer patients was 37% higher than expected when compared to the general population. A national study in Scotland showed the relative risk of suicide to be 1.5 in cancer sufferers [12]. This is especially of concern when a large proportion of cancer sufferers are surviving longer. In penile cancer, depression may exist in up to 50% of patients.

The impact of mental illness has far reaching consequences including disability, co-morbidity, suicide, reduced quality of life, lowered socioeconomic status, dependence on welfare, increase burden of carers and an increase in healthcare costs with the total economic burden estimated at over £3.5 billion per year [13].

In the UK approximately 350 patients are diagnosed with penile cancer each year. The majority will have 5 year disease specific survival of over 90% despite local recurrence [14]. Hence these long term survivors will live with the psychosocial and psychosexual effects of their treatment. Currently, little is offered in terms of screening penile cancer and general cancer patients for depression or anxiety. The NICE guidance issued in 2007 does not include any reference to such patients [15]. Psychological dysfunction in patients with penile cancer is likely to be common. It is likely to have effective, economical and acceptable treatment interventions, although measurement tools and interventions in this specific group are yet to be identified and placed in general use.

## Conclusion

This study demonstrates that penile cancer sufferers can exhibit significant psychological dysfunction, yet no standardised tools or interventional pathways are available. There is a need to identify adequate tools to measure and identify psychological and sexual dysfunction in this group of patients. Well designed multicentre studies are warranted which will lead to the development of patient pathways that enable the identification of patients who require intervention.

## Competing interests

The authors declare that they have no competing interests.

## Authors' contributions

VKS and MWL conceived the study and participated in its design. VKS and SBM performed the literature review and reviewed all articles suitable for inclusion in the study. SBM, MWL and VKS drafted the manuscript. All authors read and approved the final manuscript.

## Pre-publication history

The pre-publication history for this paper can be accessed here:


